# Assessing vitamin D's impact on pregnancy success: a predictive model for assisted reproductive technology outcomes

**DOI:** 10.3389/frph.2025.1510484

**Published:** 2025-02-18

**Authors:** Songwei Jiang, Zushun Chen, Liuming Li

**Affiliations:** ^1^ Reproductive Center, The First Affiliated Hospital of Guangxi Medical University, Nanning, China; ^2^ Department of Hepatobiliary Surgery, Guangxi Medical University Cancer Hospital, Nanning, China

**Keywords:** infertility, ART, vitamin D, factors of affecting pregnancy, logistic regression analysis

## Abstract

**Objective:**

To investigate the correlation between vitamin D levels and clinical pregnancy rates in infertile women undergoing *in vitro* fertilization (IVF)/intracytoplasmic sperm injection (ICSI) procedures and to assess the utility of vitamin D levels in developing a predictive model for assisted reproductive technology (ART) outcomes.

**Methods:**

A total of 188 infertile patients receiving their initial IVF or ICSI treatment at our reproductive center between June 2020 and July 2021 were selected for data collection. Vitamin D levels and other relevant ART-related factors were used to construct a predictive model.

**Result:**

The multivariate regression analysis revealed that several independent variables significantly impacted ART pregnancy outcomes, including infertility age, vitamin D level, reproductive anti-Müllerian hormone, antral follicle count, Gn dose, daily endometrial thickness after human chorionic gonadotropin (HCG) administration, and number of retrieved eggs. The area under the receiver operating characteristic curve for this comprehensive model was 75.34%, with a standard error of 0.045 and *p*-value of 0.003 (95% confidence interval 0.712–0965). Furthermore, the multivariate regression analysis identified specific independent variables that might influence vitamin D levels, such as the number of embryos obtained, daily endometrial thickness after HCG administration, and clinical pregnancy.

**Conclusion:**

The developed predictive model integrating serum 25-Hydroxyvitamin D level and ART-related factors holds significant clinical value in forecasting pregnancy outcomes.

## 1 Introduction

Infertility impacts approximately 15% of couples globally, with treatments such as *in vitro* fertilization (IVF) and intracytoplasmic sperm injection (ICSI) offering hope yet yielding variable success rates. Although assisted reproductive technology (ART) outcomes are influenced by factors like ovarian reserve, measured through antral follicle count (AFC) and anti-Müllerian hormone (AMH) levels, recent studies suggest a significant role of vitamin D in reproductive health (1, 2). Vitamin D, primarily known for its role in bone metabolism, is increasingly recognized as a multifunctional hormone influencing various physiological processes, including reproduction (3). Vitamin D receptors (VDR) are widely expressed in reproductive tissues, such as the ovaries, endometrium, and placenta, indicating its involvement in key reproductive processes like follicular development, oocyte maturation, and endometrial receptivity (4). Notably, vitamin D deficiency is prevalent among women of reproductive age and has been linked to adverse reproductive and metabolic outcomes, including polycystic ovary syndrome (PCOS) and poor ART outcomes (5–7). Research shows conflicting results on whether vitamin D levels correlate with improved ART success, indicating a need for further investigation (8–10).

The aim of the present study was to assess the relationship between serum vitamin D levels and clinical pregnancy rates in women undergoing IVF/ICSI. By exploring the potential of vitamin D levels alongside established predictors like AMH and AFC, we sought to enhance predictive models for ART outcomes, facilitating more personalized treatment approaches.

## 2 Methods

### 2.1 Study design and participants

This was a retrospective cohort study conducted at the Guangxi Medical University First Affiliated Hospital Reproductive Center. We enrolled 188 infertile patients who underwent routine IVF and ICSI treatments between June 2020 and July 2021. Patients were categorized into two groups based on clinical pregnancy outcomes: those who achieved successful pregnancy and those who did not. This study was approved by the Institutional Review Board of Guangxi Medical University First Affiliated Hospital (project number: IRB2020-1234, date of approval: 1 May 2020). All methods were performed in accordance with the Declarations of Helsinki, and written informed consent was provided by all patients before the study.

### 2.2 Inclusion and exclusion criteria

The inclusion criteria were as follows: patients who underwent fresh embryo transfers, complete follow-up records, no history of genetic diseases in either partner, no uterine abnormalities or concurrent adenomyosis in female patients, all participants were part of a controlled ovulation induction program, and none had taken or injected vitamin D-related preparations within the previous 6 months.

### 2.3 Exclusion criteria included

The exclusion criteria were as follows: cancellation of the embryo transfer for any reason; recipients of a frozen embryo transfer; patients with incomplete follow-up; and the presence of systemic diseases, such as diabetes, kidney disease, hypertension, or immune system disorders.

### 2.4 Data collection

Comprehensive data were collected, including baseline demographic and clinical characteristics [age, body mass index (BMI), duration of infertility, type of infertility], biochemical markers (AMH, AFC, and basal hormone levels including FSH, LH, E2, P, PRL, T, DHEA), treatment details (COH protocol, total Gn dosage, duration of Gn administration), and outcomes (endometrial thickness on the day of human chorionic gonadotropin (HCG) administration, E2 levels on HCG day, number of oocytes retrieved, number of embryos obtained, clinical pregnancy outcome).

### 2.5 Laboratory methods

Vitamin D levels were measured using a chemiluminescence immunoassay (CLIA). All hormonal measurements were performed in the hospital's central laboratory under standardized conditions using Elecsys Vitamin D Total Assay Kit (Roche Diagnostics, USA) and Access AMH Assay Kit (Coulter Beckman, USA).

### 2.6 Statistical analysis

Data were analyzed using SPSS software version 20.0. Continuous variables were expressed as mean ± standard deviation or median where appropriate, and categorical variables were expressed as percentages. The differences between groups were assessed using Student's *t*-test for normally distributed data and the Mann–Whitney *U*-test for non-normally distributed data. Chi-square or Fisher's exact test was used for categorical data. Logistic regression analysis was employed to identify independent predictors of clinical pregnancy. A *p*-value <0.05 was considered statistically significant.

## 3 Results

### 3.1 Demographic and clinical characteristics of clinical pregnancies

The overall clinical pregnancy rate for IVF/ICSI clinical pregnancies was 37.8% (71/188). There was no statistically significant difference between the successful and unsuccessful pregnancy groups in terms of BMI, type of infertility, basal sex hormones bFSH, bLH, bE2, P, PRL, and T, number of Gn days, HCG day E2 (estrogen value), method of fertilization (IVF/ICSI), and oocyte-stage embryos (*p* > 0.05) (Table 1). In contrast, patients in the pregnancy group were significantly younger (mean age 29.89 ± 4.03 years) compared to the non-pregnancy group (mean age 32.42 ± 5.11 years; *p* = 0.026) and had shorter infertility durations (mean time 4.53 ± 2.15 vs. 5.32 ± 2.31 years; *p* = 0.011). These findings align with those of prior studies suggesting that advanced age and prolonged infertility negatively impact ART outcomes, likely due to declining ovarian reserve and reduced oocyte quality. Higher mean levels of AFC (17.25 ± 5.98 vs. 13.25 ± 6.62; *p* = 0.009) and AMH (3.68 ± 2.58 vs. 2.99 ± 1.41; *p* = 0.04) in the pregnancy group further support the importance of robust ovarian reserve in achieving successful ART outcomes. In addition, mean endometrial thickness on the day of HCG administration was greater in the pregnancy group (12.63 ± 2.09 mm vs. 10.52 ± 2.54 mm; *p* = 0.035) and mean vitamin D levels were higher (29.77 ± 13.3 vs. 24.97 ± 11.16 µg/L; *p* = 0.03), suggesting enhanced endometrial receptivity and potentially improved implantation potential.

**Table 1:** Demographic and clinical characteristics of clinical pregnancies conceived through ART.

Variable	Pregnancy group (71)	Non-pregnancy group (117)	*p*
Age (years)	29.89 ± 4.03	32.42 ± 5.11	0.026*
BMI (kg/m^2^)	24.18 ± 2.98	24.37 ± 3.39	0.131
Infertility duration (years)	4.53 ± 2.15	5.32 ± 2.31	0.011*
Infertility type			
Primary	45 (61.4%)	76 (65.0%)	0.236
secondary	26 (36.6%)	41 (35.0%)	0.421
AFC	17.25 ± 5.98	13.25 ± 6.62	0.009*
Vitamin D levels (μg)	29.77 ± 13.3	24.97 ± 11.16	0.03*
AMH (ng/ml)	3.68 ± 2.58	2.99 ± 1.41	0.04*
Basic sex hormone levels			
bFSH (mIU/ml)	6.92 ± 1.32	7.63 ± 1.01	0.032*
bLH (mIU/ml)	6.12 ± 2.16	5.98 ± 3.24	0.661
E_2_ (pg/ml)	33.58 ± 14.42	34.89 ± 18.23	0.534
P (ng/ml)	0.86 ± 0.09	0.88 ± 0.08	0.732
PRL (ng/ml)	14.62 ± 8.77	14.92 ± 9.75	0.552
T (ng/dl)	0.36 ± 0.13	0.41 ± 0.15	0.085
DHEA	10.41 ± 6.32	9.36 ± 1.25	0.019*
COH type			
GnRH long regimen	42 (59.2%)	79 (67.5%)	0.028*
GnRH antagonist regimen	29 (40.8%)	32 (27.4%)	0.012*
Total amount of Gn	1,728.88 ± 788.45	1,833.67 ± 613.50	0.034*
Gn days	10.85 ± 3.21	10.77 ± 4.56	0.225
Fertilization mode			0.081
IVF	46 (64.8%)	82 (70.1%)	0.0635
ICSI	19 (26.8%)	31 (26.5%)	0.236
Half-ICSI	6 (8.5%)	4 (3.4%)	0.136
Number of pregnancies	1.12 ± 0.13	2.04 ± 0.35	0.019*
Endometrial thickness on the day of HCG	12.63 ± 2.09	10.52 ± 2.54	0.035*
HCG day E_2_	2,053.21 ± 186.14	1,967.52 ± 204.14	0.474
Number of eggs obtained	15.52 ± 4.68	13.44 ± 5.99	0.023*
Number of embryos obtained	5.77 ± 0.46	4.64 ± 0.51	0.062

* *p* < 0.05.

### 3.2 Independent factors influencing clinical pregnancy

To better understand the independent factors influencing ART pregnancy success, a stepwise backward logistic regression analysis was conducted. As presented in Table 2, seven independent predictors of clinical pregnancy were identified. These included age [β = 0.0921, odds ratio (OR) 1.08, 95% confidence interval (CI) 0.87–1.34; *p* = 0.016], AFC (β = 1.0652, OR 2.51, 95% CI 2.00–4.93; *p* = 0.033), vitamin D levels (β = 0.3758, OR 1.46, 95% CI 1.04–2.03; *p* = 0.027), AMH (β = 0.1697, OR 2.03, 95% CI 1.63–3.21; *p* = 0.037), Gn dose (β = 0.3296, OR 1.26, 95% CI 1.02–1.55; *p* = 0.030), endometrial thickness on HCG day (β = 1.6201, OR 1.97, 95% CI 1.28–2.04; *p* = 0.019), and the number of eggs retrieved (β = 1.5634, OR 1.44, 95% CI 0.48–1.62; *p* = 0.035).

**Table 2:** Multifactorial logistic regression analysis related to pregnancy.

Variable	β	SE	OR	95% CI	*p*
Age (years)	0.0921	0.1102	1.08	0.87–1.34	0.016*
AFC	1.0652	0.1136	2.51	2.00–4.93	0.033*
Vitamin D levels (μg)	0.3758	0.1697	1.46	1.04–2.03	0.027*
AMH (ng/ml)	0.1697	0.1224	2.03	1.63–3.21	0.037*
Basic sex hormone levels					
bFSH (mIU/ml)	0.9211	0.1254	1.04	1.35–3.67	0.358
DHEA	1.1021	0.2514	1.69	1.24–2.31	0.085
COH regimen					
GnRH long regimen	1.3684	0.3511	2.03	0.36–1.05	0.506
GnRH antagonist regimen	0.8564	0.3222	2.98	1.60–5.65	0.141
Total amount of Gn	0.3296	0.3621	1.26	1.02–1.55	0.030*
Number of pregnancies	1.1141	0.2536	1.85	0.98–1.85	0.194
Endometrial thickness on the day of HCG	1.6201	0.1024	0.68	1.28–2.04	0.019*
Number of eggs obtained	1.5634	0.1121	0.97	0.48–1.62	0.035*

OR, odds ratio; CI, confidence interval; β, estimation parameters of regression models; SE, standard error of the regression models.

* *p* < 0.05.

Notably, each unit increased in vitamin D levels was associated with a 46% higher likelihood of clinical pregnancy, emphasizing its potential role in improving ART outcomes. Similarly, higher AFC and AMH levels strongly predicted pregnancy success, reflecting the critical role of ovarian reserve in achieving positive ART outcomes. In addition, greater endometrial thickness on the day of HCG and a higher number of eggs retrieved were positively associated with clinical pregnancy, suggesting the importance of endometrial receptivity and effective ovarian stimulation.

### 3.3 Predictive modeling of influencing factors associated with clinical pregnancy

A full-variable logistic regression model was constructed to predict the probability of clinical pregnancy, with clinical pregnancy status as the dependent variable. The independent variables included factors that were significantly associated with pregnancy outcomes in previous analyses: age, AFC, vitamin D level, AMH, Gn dose, endometrial thickness on the day of HCG administration, and the number of eggs retrieved (Table 3). The predictive model for the probability of clinical pregnancy was expressed as: *P* = 1/(1 + y), where y = exp[−(3.538–0.0733  ×  age + 0.2962 × AFC + 0.4214 × vitamin D + 1.3624 × AMH + 0.2287 × Gn dosage + 0.2698 × endometrial thickness + 0.3241 × number of eggs retrieved)].This model integrates key clinical and biochemical factors to estimate the likelihood of clinical pregnancy in ART patients. It underscores the importance of combining age, ovarian reserve markers (AFC, AMH), vitamin D levels, and endometrial receptivity metrics to improve outcome prediction. The inclusion of vitamin D in the model highlights its potential role as a modifiable factor in enhancing ART success.

**Table 3:** Regression model of factors associated with infertility.

Variable	β	SE	OR	95% CI	*p*
Age (years)	0.0733	0.1311	1.23	0.95–2.14	0.011*
AFC	0.2962	0.1421	2.14	1.35–3.26	0.037*
Vitamin D levels (μg)	0.4214	0.2684	2.63	0.65–1.92	0.027*
AMH (ng/ml)	1.3624	0.1651	3.97	0.79–2.10	0.3758
bFSH (mIU/ml)	0.1697	0.3321	4.25	1.60–5.65	0.4428
DHEA	2.3541	0.1241	0.58	0.95–3.65	0.321
GnRH long regimen	0.1774	0.3688	1.93	2.35–6.34	0.506
GnRH antagonist regimen	1.1000	0.1532	3.00	1.35–3.94	0.958
Total amount of Gn	0.2287	0.3263	2.68	0.95–2.57	0.032*
Number of pregnancies	0.8792	0.2547	3.82	0.84–2.15	0.381
Endometrial thickness on the day of HCG	0.2698	0.1698	1.98	2.04–4.32	0.020*
Number of eggs obtained	0.3241	0.1698	1.22	0.66–2.11	0.042*

β, estimation parameters of regression models; SE, the standard error of regression models

* *p* < 0.05.

### 3.4 Establish the model to fit the superiority receiver operating characteristic (ROC) curve and evaluate the validity of the equation

**Figure 1:**
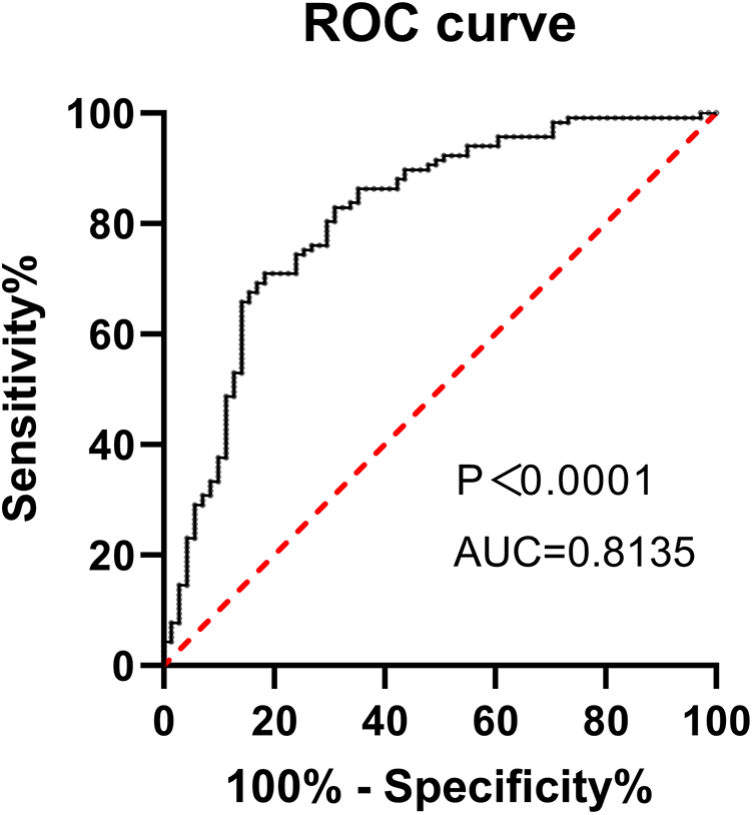
ROC curve for the predictive model of ART success.

The predicted probability of clinical pregnancy was calculated using a regression model compared with actual clinical pregnancy outcomes to generate a ROC curve, determining the area under the ROC curve (AUC). The AUC of the model was 75.34% (standard error 0.045, 95% CI 0.712–0.965; *p* = 0.003), which indicated that the effectiveness of the logistic prediction model was good (Figure 1).

### 3.5 Basic information about different vitamin D groups

Among the 188 cases included in this study, 116 were classified into the vitamin D non-deficient group (vitamin D >50 nmol/L) and 72 were categorized into the vitamin D-deficient group. There were no significant differences between the two groups among baseline characteristics, including age, BMI, duration of infertility, type of infertility, ovarian reserve markers (AFC, AMH), basal sex hormone levels, COH regimens, Gn dosage, or fertilization methods (*p* > 0.05) (Table 4).

**Table 4:** Comparison of basic information and pregnancy outcome in different vitamin D groups.

Variable	Vitamin D non-deficient group (*n* = 116)	Vitamin D deficiency group (*n* = 72)	*p*
Age (years)	28.73 ± 3.054	28.58 ± 3.670	0.472
BMI	21.56 ± 1.84	21.56 ± 1.60	0.131
Infertility duration (years)	3.05 ± 2.29	3.67 ± 2.27	0.451
Infertility type			0.593
Primary	45 (38.8%)	30 (41.7%)	
Secondary	71 (61.2%)	42 (58.3%)	
AMH (ng/ml)	3.78 ± 2.58	3.62 ± 2.56	0.178
AFC	16.35 ± 3.52	15.71 ± 3.54	0.094
Basic sex hormone levels			
bFSH (mIU/ml)	6.77 ± 1.74	7.01 ± 1.21	0.124
bLH (mIU/ml)	6.12 ± 2.16	5.98 ± 3.24	0.661
E_2_ (pg/ml)	33.58 ± 24.42	34.89 ± 28.23	0.214
P (ng/ml)	0.86 ± 0.93	0.88 ± 1.18	0.158
PRL (ng/ml)	14.62 ± 8.77	14.92 ± 9.75	0.367
T (ng/dl)	0.42 ± 0.14	0.39 ± 0.12	0.442
DHEA	8.56 ± 1.91	9.01 ± 1.78	0.115
COH type			0.854
GnRH long regimen	79 (68.1)	49 (42.2%)	
GnRH antagonist regimen	37 (31.9%)	23 (31.9%)	
Dose of Gn	2,381.55 ± 589.14	2,401.21 ± 601.23	0.316
Gn days	14.16 ± 5.11	13.25 ± 4.04	0.077
Total amount of Gn			0.234
<1,808.01	84 (72.4%)	47 (65.3%)	
≥1,808.01	32 (27.6%)	25 (34.7%)	
Gn days	10.2 ± 2.69	11.1 ± 3.21	0.771
Fertilization mode			0.158
IVF	62 (53.4%)	39 (54.2%)	
ICSI	44 (37.9%)	28 (38.9%)	
Half-ICSI	10 (8.62%)	5 (6.94%)	
Number of pregnancies	1.56 ± 0.13	1.84 ± 0.20	0.115
Endometrial thickness on the day of HCG	11.97 ± 1.65	10.07 ± 1.01	0.036*
HCG day E_2_	2,131.51 ± 218.42	1,953.14 ± 221.11	0.354
Number of eggs obtained	15.68 ± 5.32	14.35 ± 4.65	0.097
Number of embryos obtained	5.36 ± 0.77	4.88 ± 0.97	0.029*
Clinical pregnancy	49 (42.2%)	22 (30.6%)	0.015*

* *p* < 0.05.

In contrast, several ART outcome indicators showed statistically significant differences between the two groups. Endometrial thickness on the day of HCG administration was significantly higher in the vitamin D non-deficient group compared to the deficient group (11.97 ± 1.65 mm vs. 10.07 ± 1.01 mm; *p* = 0.036), indicating better endometrial receptivity in patients with sufficient vitamin D levels. In addition, the number of embryos obtained was greater in the non-deficient group (5.36 ± 0.77 vs. 4.88 ± 0.97; *p* = 0.029), and the clinical pregnancy rate was significantly higher in the vitamin D non-deficient group (42.2% vs. 30.6%; *p* = 0.015). These findings suggest that sufficient vitamin D levels may improve ART outcomes by enhancing both endometrial receptivity and embryo quality.

### 3.6 Logistic regression analysis of factors associated with vitamin D affecting pregnancy outcome

As shown in Table 5, three factors associated with vitamin D levels were significantly related to pregnancy outcomes: the number of embryos obtained (β = 0.5532, OR 0.53, 95% CI 0.31–0.95; *p* = 0.024), endometrial thickness on the day of HCG administration (β = 0.2354, OR 1.97, 95% CI 0.44–1.68; *p* = 0.022), and clinical pregnancy (β = 0.3695, OR 0.55, 95% CI 0.33–1.24; *p* = 0.019). Patients with higher vitamin D levels had a significantly greater number of embryos obtained, as indicated by the positive association in the regression analysis. Similarly, endometrial thickness on the day of HCG was significantly greater in the vitamin D non-deficient group, suggesting a correlation between sufficient vitamin D levels and improved endometrial receptivity. Furthermore, vitamin D sufficiency was significantly associated with clinical pregnancy, highlighting its role in promoting implantation success.

**Table 5:** Factors related to vitamin D influencing pregnancy outcome by logistic regression analysis.

Variable	β	SE	OR	OR (95% CI)	*p*
Age (years)	0.8574	0.1487	1.25	0.12–0.98	0.591
BMI	1.2114	0.1214	1.36	0.65–2.11	0.782
Infertility duration (years)	0.5841	0.0952	1.22	0.35–0.87	0.152
Infertility type	0.6234	0.0854	0.98	0.12–1.09	0.098
AMH	1.0541	0.0741	0.35	0.57–1.62	0.085
AFC	0.6241	0.0951	0.85	0.84–2.35	0.264
Basic sex hormone levels	1.2413	0.0814	0.47	0.63–1.94	0.152
bFSH (mIU/ml)	0.5321	0.1147	1.23	0.97–2.47	0.634
bLH (mIU/ml)	0.6471	0.1024	0.69	0.68–1.96	0.095
E2 (pg/ml)	1.2141	0.1234	0.58	0.52–2.14	0.114
*P* (ng/ml)	1.6871	0.1475	0.47	0.48–2.61	0.357
PRL (ng/ml)	0.3657	0.1357	0.36	0.52–2.01	0.634
T (ng/dl)	0.6304	0.1224	0.85	0.36–1.63	0.287
DHEA	0.9854	0.1123	0.89	0.45–1.53	0.741
COH type	0.3624	0.1478	0.47	0.42–2.08	0.167
Total amount of Gn	0.3624	0.0952	0.98	0.33–1.24	0.074
Gn days	0.9874	0.1874	0.58	0.54–2.03	0.195
Dose of Gn					
<1,808.01	0.6214	0.1474	0.98	0.50–2.05	0.510
≥1,808.01	1.2414	0.2141	1.22	0.57–1.85	0.447
Gn days	1.3547	0.1470	0.34	0.63–2.57	0.097
Fertilization mode					
IVF	0.2141	0.1254	0.65	0.47–1.28	0.735
ICSI	0.3314	0.1587	0.25	0.84–2.69	0.665
Half-ICSI	0.5241	0.1147	0.63	0.63–1.99	0.497
Number of pregnancies	0.7484	0.0987	0.57	0.74–2.65	0.082
Endometrial thickness on the day of HCG	0.2354	0.1374	1.97	0.44–1.68	0.022*
HCG day E_2_	0.3698	0.2414	1.22	0.74–1.87	0.192
Number of eggs obtained	0.1987	0.1547	1.14	0.58–1.98	0.095
Number of embryos obtained	0.5532	0.0921	0.53	0.31–0.95	0.024*
Clinical pregnancy	0.3695	0.0913	0.55	0.33–1.24	0.019*

HR, hazard ratio; CI, confidence interval; β, estimation parameters of regression models; SE, standard error of regression models.

* *p* < 0.05.

## 4 Discussion

This study demonstrates that adequate vitamin D levels are significantly associated with improved ART outcomes, including higher clinical pregnancy rates and better ovarian reserve markers. Patients with sufficient vitamin D levels showed significantly higher AFC and AMH levels, which are crucial predictors of ART success. In addition, vitamin D sufficiency correlated with increased endometrial thickness on the day of HCG administration, a key factor for implantation. Our predictive model, incorporating vitamin D alongside traditional reproductive parameters, achieved an area under the ROC curve of 75.34%, emphasizing its potential as a biomarker for ART success. These findings highlight the multifaceted role of vitamin D in enhancing ovarian response, improving endometrial receptivity, and supporting clinical pregnancy in women undergoing IVF or ICSI.

The relationship between vitamin D and infertility has been widely studied, with growing evidence supporting its critical role in reproductive health (11, 12). Our findings align with those of prior research, demonstrating that adequate vitamin D levels are associated with higher clinical pregnancy rates, better ovarian reserve markers, and improved endometrial receptivity. Specifically, patients in our study with adequate vitamin D levels had significantly greater endometrial thickness, a crucial factor for implantation, and higher AFC and AMH levels, reflecting improved ovarian reserve. These results support findings by Kolcsar et al., who reported that serum 25-hydroxyvitamin D levels positively influence mid-luteal progesterone (13), suggesting that vitamin D supports ovulation and corpus luteum function, both essential for successful implantation. Similarly, Chu et al. demonstrated that vitamin D enhances endometrial receptivity, consistent with our findings of increased endometrial thickness in vitamin D-sufficient patients (14). However, despite these positive associations, controversies remain regarding the role of vitamin D across different infertility etiologies. For instance, Grzeczka et al. suggested that serum vitamin D levels above 20 ng/ml improve ART outcomes, particularly in women with endocrine-related infertility such as PCOS (15). Conversely, Varbiro et al. found no significant association between vitamin D and ART outcomes in non-PCOS patients, emphasizing that baseline vitamin D levels and individual factors may influence its effectiveness (16). Our study similarly highlights the need for identifying specific subpopulations, such as women with unexplained infertility or endocrine disorders, who may benefit most from vitamin D supplementation. This is particularly important as the effect of vitamin D may vary depending on baseline characteristics, as reflected by the variable findings in previous studies.

Vitamin D's beneficial effects on ART outcomes may be mediated through its receptor expression in reproductive tissues, including the ovaries, endometrium, and placenta. Our study demonstrated a significant association between vitamin D sufficiency and increased endometrial thickness, supporting its role in enhancing endometrial receptivity, a critical factor for successful implantation. This aligns with prior findings by Ozkan et al., who reported that vitamin D promotes cellular differentiation and reduces inflammation in the endometrium (12). In addition, the observed correlation between vitamin D levels and ovarian reserve markers (AFC and AMH) in our study suggests that vitamin D may directly influence ovarian follicle development and function. This is consistent with studies by Voulgaris et al., which highlighted the role of vitamin D in regulating the hypothalamic-pituitary-gonadal (HPG) axis (17), thereby improving gonadal function and hormonal regulation necessary for follicular recruitment. Moreover, our findings of increased clinical pregnancy rates in vitamin D-sufficient patients may reflect vitamin D's immunomodulatory effects (18). Thompson et al. demonstrated that vitamin D reduces uterine natural killer (NK) cell activity and inflammatory cytokine production, creating a more favorable environment for embryo implantation (19). Our study reinforces this mechanism, as patients with sufficient vitamin D levels not only showed improved endometrial receptivity but also achieved higher clinical pregnancy rates, underscoring the multifaceted role of vitamin D in supporting ART success.

### 4.1 Limitations and future directions

Despite these promising findings, our study has several limitations. First, the single-center, retrospective design may restrict the generalizability of our results to other populations and clinical settings. Larger, multicenter prospective studies are necessary to validate our predictive model and ensure its applicability across diverse populations (11). Second, seasonal variation in serum 25(OH)D levels was not accounted for in our study. Vitamin D levels are known to fluctuate with seasonal sun exposure, which could have influenced the baseline vitamin D status of participants. For example, patients evaluated during winter months may have had lower serum 25(OH)D levels compared to those assessed in summer, potentially introducing variability in our results (20). Future studies should incorporate seasonal data to control for this factor and determine whether seasonal fluctuations impact ART outcomes. Third, although we analyzed vitamin D levels, the lack of comprehensive nutritional data, such as dietary intake and overall nutritional status, limits our ability to assess the broader impact of nutrition on ART outcomes. This limitation is particularly relevant given that overall nutrition, alongside vitamin D levels, plays a crucial role in reproductive health (8–10). In addition, interventional studies evaluating the effects of vitamin D supplementation on ART success are crucial to establish causality, determine optimal dosing protocols, and identify subpopulations of patients who may benefit most from supplementation. Furthermore, our findings suggest that vitamin D impacts both ovarian reserve and endometrial receptivity, emphasizing the need to investigate its genetic and molecular pathways in reproductive tissues. Finally, exploring interactions between vitamin D and other nutritional or hormonal factors may help refine holistic approaches to reproductive health, ultimately improving ART outcomes.

In conclusion, our study enhances the understanding of vitamin D's role in ART success and introduces a predictive model that incorporates both traditional and novel predictors of clinical pregnancy. It encourages a shift toward more holistic treatment paradigms in reproductive medicine, where nutritional and hormonal factors are considered in concert to optimize patient outcomes.

## Data Availability

The raw data supporting the conclusions of this article will be made available by the authors, without undue reservation.
